# All-carbon [3 + 2] cycloaddition in natural product synthesis

**DOI:** 10.3762/bjoc.16.251

**Published:** 2020-12-09

**Authors:** Zhuo Wang, Junyang Liu

**Affiliations:** 1 School of Medicine, Southern University of Science and Technology, Shenzhen, 518055, People’s Republic of China; 2 Academy for Advanced Interdisciplinary Studies, Southern University of Science and Technology, Shenzhen, 518055, People’s Republic of China

**Keywords:** all-carbon, cyclization, [3 + 2] cycloaddition, natural product synthesis, stereocenters

## Abstract

Many natural products possess interesting medicinal properties that arise from their intriguing chemical structures. The highly-substituted carbocycle is one of the most common structural features in many structurally complicated natural products. However, the construction of highly-substituted, stereo-congested, five-membered carbocycles containing all-carbon quaternary center(s) is, at present, a distinct challenge in modern synthetic chemistry, which can be accessed through the all-carbon [3 + 2] cycloaddition. More importantly, the all-carbon [3 + 2] cycloaddition can forge vicinal all-carbon quaternary centers in a single step and has been demonstrated in the synthesis of complex natural products. In this review, we present the development of all-carbon [3 + 2] cycloadditions and illustrate their application in natural product synthesis reported in the last decade covering 2011–2020 (inclusive).

## Introduction

**Figure 1:**
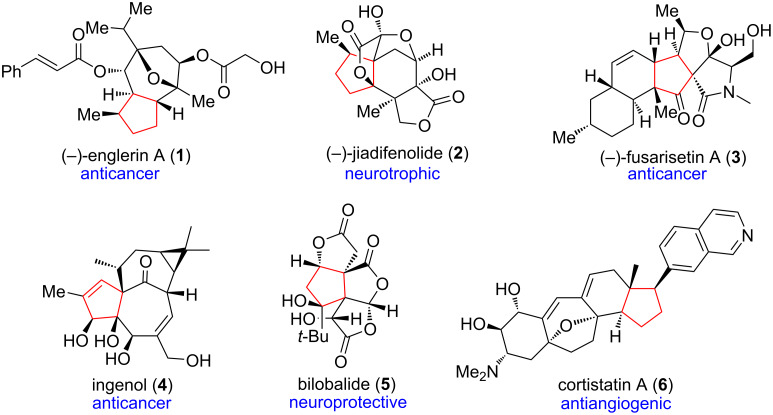
Highly-substituted five-membered carbocycle in biologically significant natural products.

**Figure 2:**
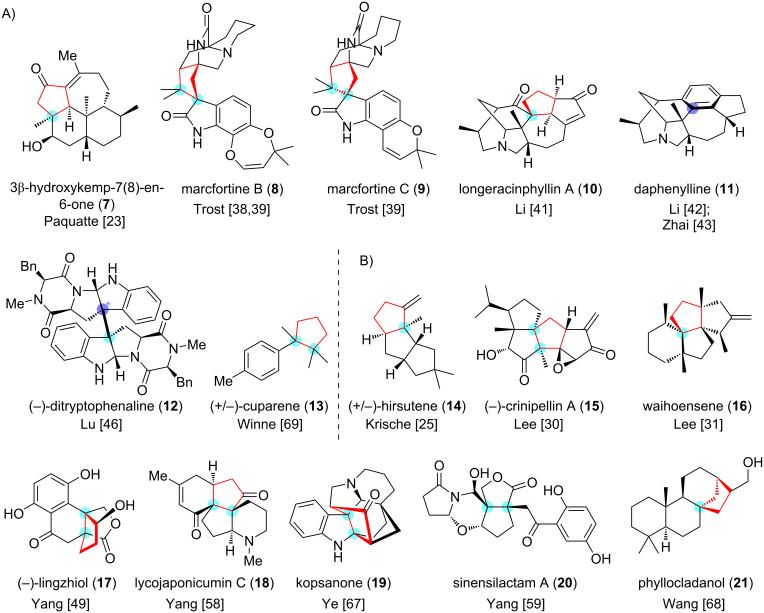
Natural product synthesis featuring the all-carbon [3 + 2] cycloaddition. (Quaternary carbon center(s) created by all-carbon [3 + 2] cyclization are highlighted in cyan; quaternary carbon center(s) created that are removed by subsequent transformations are highlighted in lilac; cyclopentane structures forged by the all-carbon [3 + 2] cyclization are labeled in red). (A) The intermolecular all-carbon [3 + 2] cyclization features as the key reaction. (B) The intramolecular all-carbon [3 + 2] cycloaddition features as the key reaction.

The highly-substituted, stereo-congested, five-membered carbocycle containing contiguous stereocenters is one of the most common structural features in many structurally complicated, biologically important natural products [1–7] (Figure 1). Meanwhile, the construction of quaternary carbon stereocenter(s) is, at present, a distinct challenge in modern synthetic chemistry [8–11]. Therefore, the synthesis of highly-substituted five-membered carbocycles bearing congested arrays of stereocenters within the polycyclic framework of complex natural products usually require a sophisticated synthetic planning. This issue is not trivial because only a few strategies are available for the efficient synthesis of such an intriguing molecular architecture. More importantly, the all-carbon [3 + 2] cycloaddition can forge vicinal all-carbon quaternary centers [12] in a single-step operation and provides a direct access to various substituted five-membered carbocycles. These characteristics make the all-carbon [3 + 2] cycloaddition an appealing method and/or strategy in the synthesis of complex natural products (Figure 2).

The 1,3-dipolar cycloaddition has been well-documented and widely used for the construction of five-membered heterocycles since the 1960s [13]. However, the development of the all-carbon [3 + 2] cycloaddition, for instance, Berson’s and Little’s [3 + 2] cycloaddition through diyl trapping with an olefin [14–15] and Trost’s palladium-catalyzed trimethylenemethane cycloaddition [16], which allows the preparation of five-membered carbocycles, have been emerged since the 1970s. Thereafter, many novel and important all-carbon [3 + 2] cycloaddition reactions, such as the phosphine-catalyzed [3 + 2] cycloaddition [17], platinum-catalyzed [3 + 2] cycloaddition [18], and Rhodium-catalyzed [3 + 2] cycloaddition [12], were invented and have been extensively used in natural product synthesis in the last decade. Many reviews focusing the method development of the all-carbon [3 + 2] cycloaddition have been published [19–21]. However, there is no review effort, to the best of our knowledge, has been paid attention to the development of the all-carbon [3 + 2] cycloaddition with an emphasis on the natural product synthesis. Therefore, we are motivated to provide a timely and focused review of all-carbon [3 + 2] cycloadditions in natural product synthesis.

In this review, we present the development of the all-carbon [3 + 2] cycloaddition and discuss its application in natural product synthesis reported from 2011–2020. We begin with describing the brief history of the all-carbon [3 + 2] cycloaddition with selected natural product syntheses reported before 2011 [22–26]. Next, we discuss the synthetic methods including the proposed mechanism and/or catalytic cycle and focus on illustrative examples of natural product syntheses. Moreover, several natural product syntheses featuring all-carbon [3 + 2] annulation are elaborated. Lastly, we discuss future directions and opportunities for the all-carbon [3 + 2] cycloaddition.

## Review

**Scheme 1:**
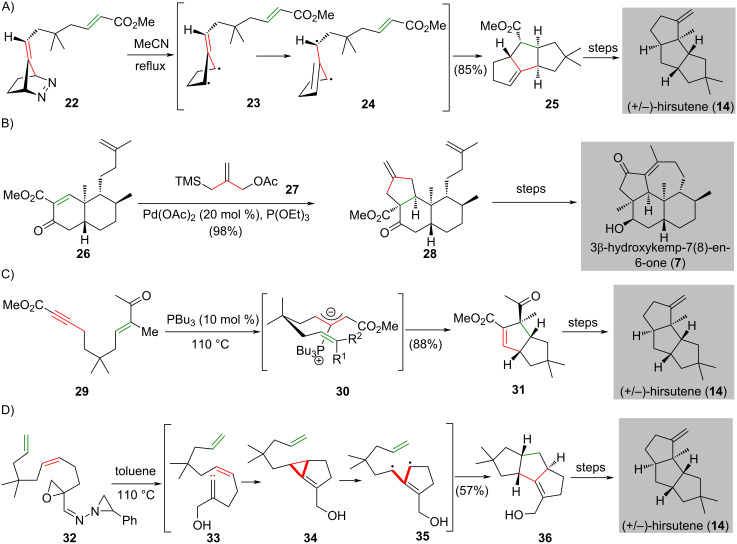
Representative natural product syntheses that feature the all-carbon [3 + 2] cyclization as the key reaction, reported before 2011. (A) TMM cycloaddition of diyl **24** resulted from dinitrogen extrusion/isomerization is used to prepare tricycle **25**, which is a synthetic precursor of (±)-hirsutene (**14**) [22]. (B) Synthesis of 3β-hydroxykemp-7(8)-en-6-one (**7**) features a palladium-catalyzed intermolecular [3 + 2] cycloaddition to generate tricycle **28** [23]. (C) A stereospecific phosphine-catalyzed [3 + 2] cycloaddition completes the synthesis of (±)-hirsutene (**14**) [25]. (D) Linear alkylidene carbenes involved TMM [3 + 2] cycloaddition produces tricycle **36** in the preparation of (±)-hirsutene (**14**) [24].

In 1981, Little and co-workers utilized a trimethylenemethane (TMM) cycloaddition as the key reaction to synthesize the tricyclic compound **25**, which led to the synthesis of (±)-hirsutene (**14**) [22] (Scheme 1A). Refluxing azo compound **22** in acetonitrile generated the proposed biradical intermediate **23** through nitrogen extrusion. This intermediate underwent isomerization to **24** and intramolecular diyl trapping through a [3 + 2] cycloaddition to give fused tricycle **25** in 85% yield. The synthesis of the hydroxykempenone 3β-hydroxykemp-7(8)-en-6-one (**7**) features Trost’s palladium-catalyzed trimethylenemethane [3 + 2] cycloaddition [27] and was reported by Paquette and co-workers in 1992 [23] (Scheme 1B). Catalytic TMM [3 + 2] cycloaddition of activated octalone **26** and the trimethylenemethane precursor **27** selectively produced adduct **28** in 98% yield, which is a synthetic precursor of 3β-hydroxykemp-7(8)-en-6-one (**7**).

Another two syntheses of (±)-hirsutene (**14**), after Little’s pioneering work [22], were accomplished by Krische [25] and Lee [24] independently in 2003. (Scheme 1C and Scheme 1D) In Krische’s synthesis, a stereospecific intramolecular phosphine-catalyzed [3 + 2] cycloaddition of 2-butynoate with electron-deficient alkene **29** afforded cycloadduct **31** in 88% yield as a single diastereomer [25] (Scheme 1C). Later, Lee`s synthesis of (±)-hirsutene (**14**) used an alkylidene carbene as source of TMM diyl in the intramolecular [3 + 2] cycloaddition [24] (Scheme 1D). Heating of epoxyaziridinyl imine **32** produced tricyclic compound **36** in 57% yield as a single product. The authors proposed that heating of epoxyaziridinyl imine **32** generates alkylidene carbene **33**. Transformation of **33** to TMM diyl **35** enables an intramolecular [3 + 2] cycloaddition to give the desired tricyclic product **36**.

### Trimethylenemethane (TMM) cycloaddition

**Scheme 2:**
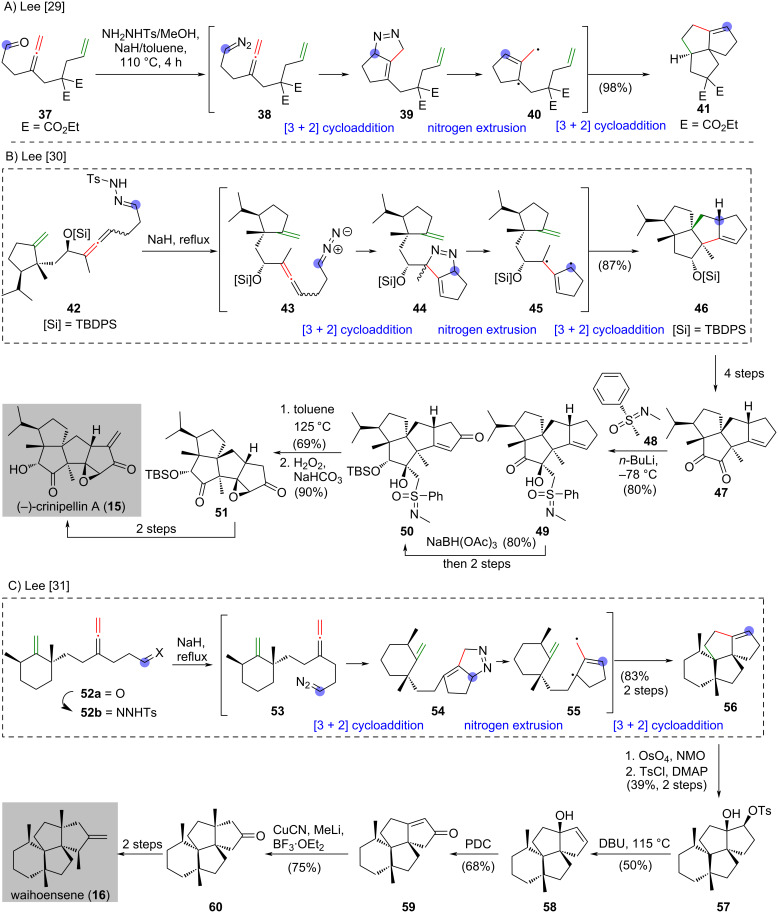
(A) An intramolecular trimethylenemethane diyl [3 + 2] cycloaddition with allenyl diazo compound **38** as a key intermediate to give angular-fused triquinane **41** [29]. (B) Synthesis of (−)-crinipellin A (**15**) [30]. (C) Synthesis of waihoensene (**16**) [31].

An intramolecular trimethylenemethane diyl [3 + 2] cycloaddition was reported by Berson [28] and Little [14] independently in the late 1970s, which was used to prepare (±)-hirsutene (**14**) in 1981 [22] (Scheme 1A). In 2003, Lee and co-workers disclosed an intramolecular trimethylenemethane diyl [3 + 2] cycloaddition with a linear alkylidene carbene as diyl source and was applied in the synthesis of linearly fused triquinane (±)-hirsutene (**14**) [24] (Scheme 1D). In 2011, the same research group used allenyl diazo compound **38**, which was generated from the reaction between aldehyde **37** and *p*-toluenesulfonehydrazide in the presence of sodium hydride upon heating, to produce diyl **40** [29] (Scheme 2A). The intramolecular trimethylenemethane diyl [3 + 2] cycloaddition of **40** led to the formation of angular fused triquinane **41** in 98% yield. The authors suggested that an intramolecular cycloaddition of the diazo group and allene **38** produces tetrahydrocyclopentapyrazole **39**. Extrusion of nitrogen from the newly formed **39** produces diyl **40**, which undergoes [3 + 2] cycloaddition to produce the angular fused triquinane **41**.

With the successful preparation of angular fused triquinane **41** by trimethylenemethane diyl [3 + 2] cycloaddition [29], enabled the synthesis of (−)-crinipellin A (**15**) [30] and waihoensene (**16**) [31] by Lee and co-workers in 2014 and 2017, respectively (Scheme 2B and Scheme 2C). The synthesis of (−)-crinipellin A (**15**) began with the treatment of hydrazone **42** with sodium hydride under reflux to produce the tetraquinane **46** in 87% yield [30] (Scheme 2B). The authors suggested that the diazo compound **43** formed undergoes an intramolecular cycloaddition to give **44**. Freshly prepared **44** was converted to diyl **45** followed by another cycloaddition to give the tetraquinane **46**. A four-step synthesis from the tetraquinane **46** gave diketone **47**. Treatment of sulfoximine **48** with *n*-butyllithium generated the corresponding anion, which selectively attacked the C-8 ketone moiety of **47** to give alcohol **49** in β-configuration in 80% yield [32]. Chemoselective and stereoselective reduction of the C-9 ketone of **49** was accomplished by treatment with NaBH(OAc)_3_ [33] and produced **50** after a two-step synthesis. Removal of the sulfoximine group in **50** upon refluxing in toluene and subsequent epoxidation afforded **51** [32], which was converted to (−)-crinipelline A (**15**) in two steps.

The synthesis of waihoensene (**16**) commenced with the conversion of aldehyde **52a** to the corresponding hydrazone **52b**, which was treated with sodium hydride under reflux to give **56** in 83% yield over two steps [31] (Scheme 2C). This transformation was rationalized as follows: freshly prepared **52b** was converted to diazo **53**, which was subjected to [3 + 2] cycloaddition to give adduct **54**. Formation of diyl **55** from **54** and subsequent [3 + 2] cycloaddition produced the tetracyclic compound **56**. Dihydroxylation of freshly prepared **56** with OsO_4_ and then selective tosylation afforded **57** in 39% yield over two steps. Exposure of **57** to DBU upon heating gave the elimination product **58**, which was subjected to an oxidative rearrangement with PDC to give enone **59** in 68% yield. Copper-mediated conjugated addition of methyllithium to enone **59** in the presence of boron trifluoride ether [34–35] produced desired ketone **60** in 75% yield. The resultant ketone **60** was converted to waihoensene (**16**) in two steps.

### Palladium-catalyzed carboxylative trimethylenemethane cycloaddition

**Scheme 3:**
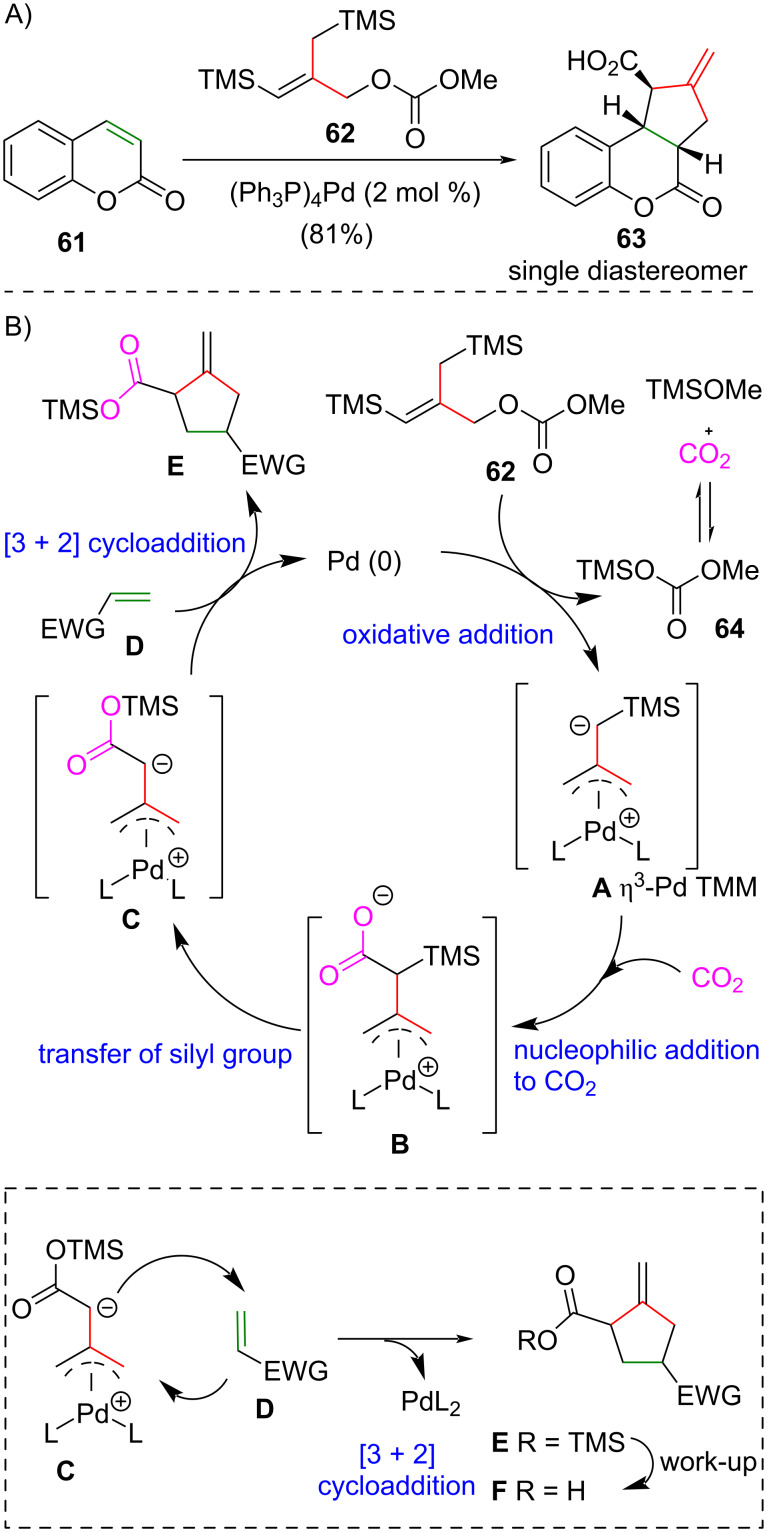
(A) Palladium-catalyzed intermolecular carboxylative TMM cycloaddition [36]. (B) The proposed mechanism.

In 1986, Trost and co-workers disclosed the palladium-catalyzed intermolecular carboxylative TMM [3 + 2] cycloaddition [36] (Scheme 3). Exposure of coumarin **61** to the silyl-substituted TMM precursor **62** in the presence of a catalytic amount of Pd(PPh_3_)_4_ afforded adduct **63** in 81% yield as a single diastereomer (Scheme 3A). Trost and co-workers proposed that the catalytic mechanism involves an oxidative addition of palladium(0) into **62** affording the η^3^-Pd TMM complex **A** [37] (Scheme 3B). Methyl trimethylsilyl carbonate (**64**) is formed as side product, which is in equilibrium with carbon dioxide and methyl trimethylsilyl ether. The electron-rich end of complex **A** attacks the carbon dioxide to give carboxylate **B**. Migration of the TMS group on carboxylate **B** generates the 1,3-dipole on **C** in the form of TMS carboxylate. An intermolecular [3 + 2] cycloaddition of **C** and alkene **D** (see Scheme 3B, inset) gives the cycloaddition adduct **E**, which is converted to the corresponding carboxylic acid (not shown) upon reaction work-up. This elegant reaction was applied in the synthesis of marcfortine B (**8**), reported by Trost and co-workers in 2007 [38] and 2013 [39].

**Scheme 4:**
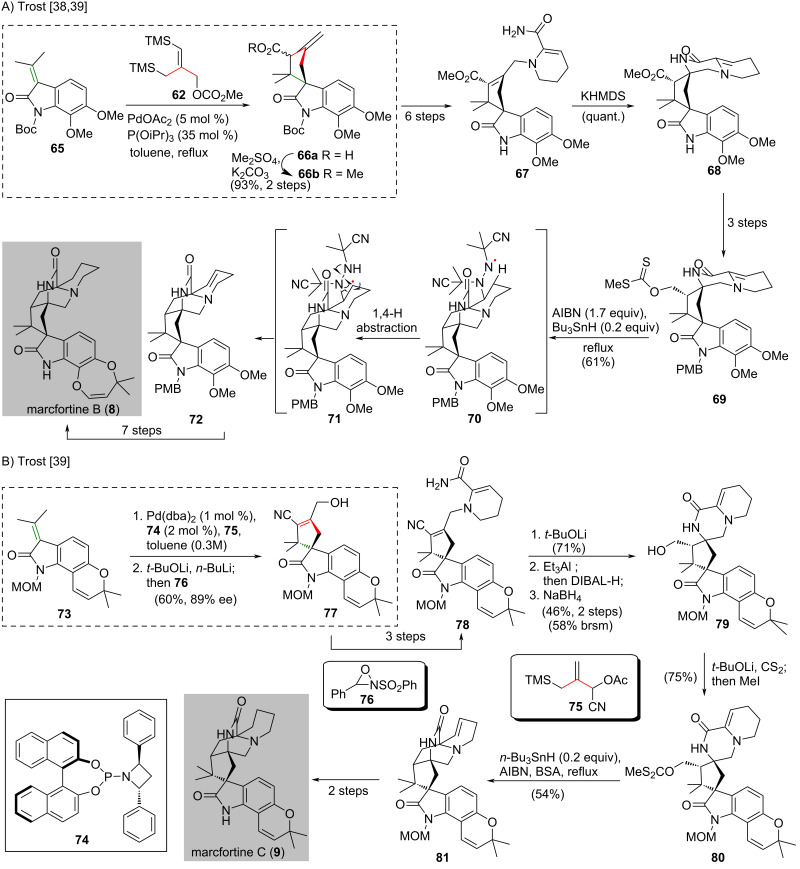
Natural product syntheses that make use of palladium-catalyzed intermolecular [3 + 2] cycloadditions of TMM. (A) Synthesis of marcfortine B (**8**) uses a palladium-catalyzed carboxylatve TMM [3 + 2] cycloaddition [38–39]. (B) Enantioselective synthesis of marcfortine C (**9**) features a palladium-catalyzed asymmetric cyano-substituted TMM [3 + 2] cycloaddition [39].

The synthesis of marcfortine B (**8**) began with palladium-catalyzed intermolecular carboxylatve TMM [3 + 2] cycloaddition [36] of enone **65** and TMM donor **62** to forge the highly-substituted spirocyclic cyclopentane **66a** [38] (Scheme 4A). Methylation of the resultant cyclopentane **66a** gave methyl ester **66b** in 93% yield over two steps. A six-step synthesis from ester **66b** gave α,β-unsaturated amide **67**, which was treated with KHMDS to facilitate an intramolecular Michael addition to give lactam **68** in quantitative yield. The conversion of freshly prepared lactam **68** to xanthante ester **69** was achieved in three steps. Exposure of xanthante ester **69** to AIBN and a catalytic amount of tributylstannane [40] led to a radical cyclization, in which the resultant alkyl radical formed was trapped by AIBN to give a proposed nitrogen-centered radical **70**. An 1,4-hydrogen abstraction of the nitrogen-centered radical on **70** produced carbon-centered radical **71**, which underwent fragmentation to afford alkene **72** in 61% yield. Marcfortine B (**8**) was synthesized from alkene **72** in seven steps.

The enantioselective synthesis of marcfortine C (**9**) commenced with a catalytic asymmetric cyano-substituted TMM cycloaddition of oxindole **73** and TMM donor **75** with Pd(dba)_2_/**74** as catalyst to give a cycloaddition adduct (not shown) [39] (Scheme 4B). Subsequent treatement with *t*-BuOLi resulted in the isomerization of the *exo*-olefin followed by exposure to *n*-butyllithium and Davis‘ oxaziridine **76** to give **77** in 60% yield with 89% ee. A three-step synthesis from **77** gave α,β-unsaturated amide **78**, which underwent successive intramolecular Michael addition and hydrolytic nitrile reduction to give **79** in 46% yield in two steps. Extensive studies of the nitrile reduction eventually identified that Et_3_Al and DIBAL-H could effectively reduce the nitrile group to the corresponding aldehyde and treatment with NaBH_4_ afforded alcohol **79**. Alcohol **79** was converted into the corresponding xanthate ester **80**. This ester **80** was exposed to an excessive amount of AIBN and *N,O*-bis(trimethylsilyl)acetamide in the presence of a catalytic amount of tributylstannane producing bicyclo[2.2.2]diazaoctane **81** in 54% yield. The authors mentioned that the employment of the previously reported conditions for the radical cyclization in the synthesis of marcfortine B (**8**) led to the decomposition of the starting material. It was suggested that the MOM group of **80** may contribute to undesired side reactions. Synthesis of marcfortine C (**9**) was accomplished from **81** in two steps.

### Phosphine-catalyzed [3 + 2] cycloaddition

**Scheme 5:**
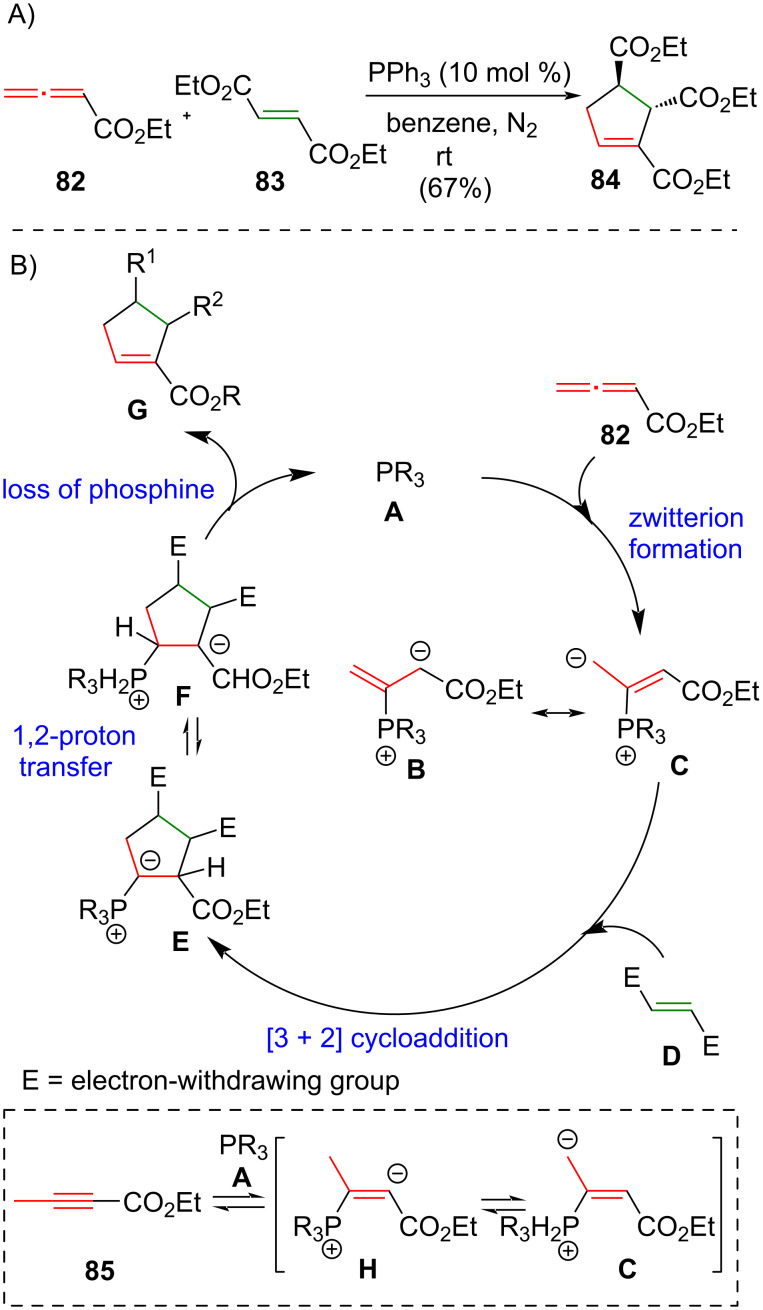
(A) Phosphine-catalyzed [3 + 2] cycloaddition [17]. (B) The proposed mechanism.

In 1995, Lu and co-workers reported a phosphine-catalyzed [3 + 2] cycloaddition, employing electron-deficient olefins and either 2,3-butadienoates or 2-butynoates to give a cyclopentene as product [17] (Scheme 5A). The reaction between ethyl 2,3-butadienoate (**82**) and diethyl fumarate (**83**) in the presence of 10 mol % of triphenylphosphine afforded *trans*-**84** in 67% yield. Under the same conditions, the use of diethyl maleate in place of diethyl fumarate (**83**) will give *cis*-**84** in 46% yield (not shown). Lu and co-workers proposed that the catalytic mechanism involves a reaction between phosphine catalyst **A** and allene **82** to give **B** and/or **C** (Scheme 5B). Catalytic [3 + 2] cycloaddition of **B** and/or **C** and alkene **D** gives the cyclic intermediates **E** and **F** in an equilibrium state through a 1,2-proton transfer. The loss of phosphine catalyst from **E** or **F** affords the cycloaddition product **G** and the catalyst is regenerated. It is noteworthy that ethyl 2-butynoate (**85**) can be used as substrate in place of ethyl 2,3-butadienoate (**82**) in the phosphine-catalyzed [3 + 2] cycloaddition. Ethyl 2-butynoate (**85**) enters the catalytic cycle by reacting with phosphine catalyst **A** to give **H** and **C**.

**Scheme 6:**
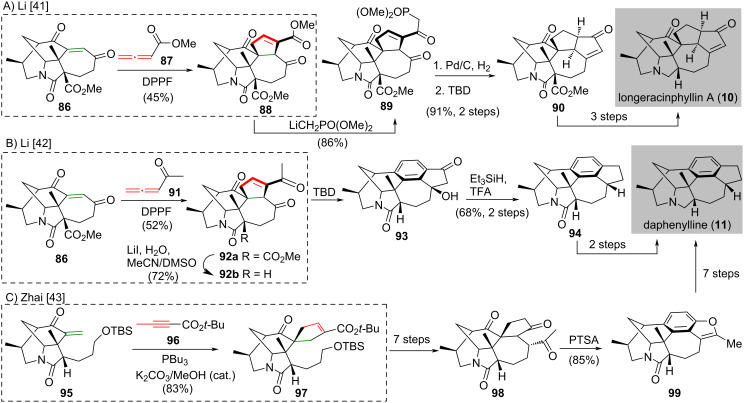
Lu’s [3 + 2] cycloaddition in natural product synthesis. (A) Synthesis of longeracinphyllin A (**10**) [41]. (B) Synthesis of daphenylline (**11**) [42]. (C) Synthesis of daphenylline (**11**) [43].

Some total syntheses of hexacyclic *Daphniphyllum* alkaloids were reported by Li’s group (longeracinphyllin A (**10**) [41] and daphenylline (**11**) [42]) and Zhai’s group (daphenylline (**11**) [43]), applying Lu’s [3 + 2] cycloaddition (Scheme 6). The synthesis of longeracinphyllin A (**10**), which was reported by Li and co-workers in 2017, used a 1,1’-bis(diphenylphosphino)ferrocene-promoted [3 + 2] cycloaddition [44] of enedione **86** and allenoate **87** to give adduct **88** in 45% yield. This adduct **88** was treated with an excess of LiCH_2_PO(OMe)_2_ to afford β-ketophosphonate **89** in 86% yield (Scheme 6A) [41]. Hydrogenation of **89** followed by an intramolecular Horner–Wadsworth–Emmons olefination produced hexacyclic enone **90** in 91% yield over two steps. The conversion of enone **90** to longeracinphyllin A (**10**) was achieved in three steps.

The syntheses of daphenylline (**11**) were reported by Li’s group [42] and Zhai’s group [43] independently in 2017 (Scheme 6B and Scheme 6C). In Li’s synthesis, the common intermediate dienone **86** was subjected to a 1,1’-bis(diphenylphosphino)ferrocene-promoted [3 + 2] cycloaddition [41] with allenyl ketone **91** to give adduct **92a** in 52% yield (Scheme 6B). This adduct **92a** underwent decarboxylation to afford **92b** in 72% yield [42]. Exposure of freshly prepared **92b** to triazabicyclodecene [45] led to a ring-expansion/aromatization/aldol cascade producing **93**, which was reduced with Et_3_SiH/TFA smoothly to give indane **94** in 68% yield over two steps. The freshly prepared indane **94** was converted to daphenylline (**11**) in two steps. The preparation of daphnipaxianine A and himalenine D (not shown) were also disclosed in the same work but are not described here.

Zhai’s synthesis of daphenylline (**11**) used Lu’s phosphine-catalyzed [3 + 2] cycloaddition [17] of enone **95** and *tert*-butyl 2-butynoate (**96**) with PBu_3_ and K_2_CO_3_/MeOH as additive to give the cycloaddition adduct **97** in 83% yield [43] (Scheme 6C). A seven-step synthesis from **97** gave pentacyclic ketone **98**. Pentacyclic ketone **98** was exposed to PTSA under reflux to give the Wagner–Meerwein rearrangement product **99** in 85% yield. The synthesis of daphenylline (**11**) was completed by a seven-step synthesis from benzofuran **99**.

### Phosphine-catalyzed enantioselective [3 + 2] annulation

**Scheme 7:**
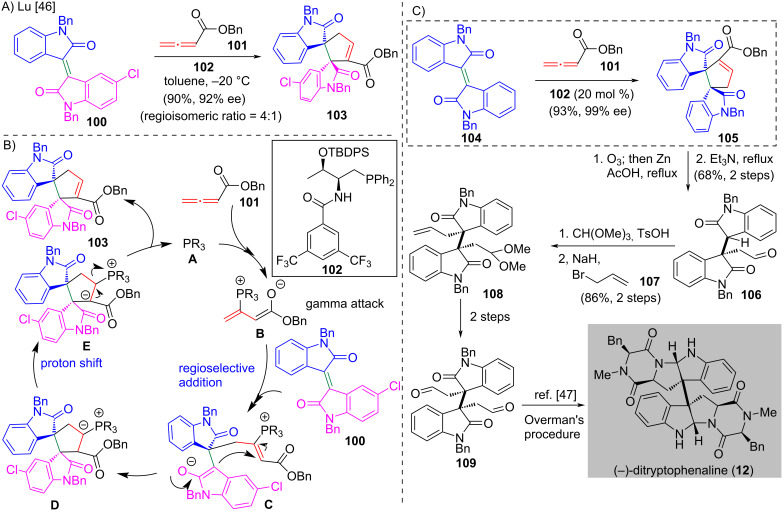
(A) Phosphine-catalyzed [3 + 2] annulation of unsymmetric isoindigo **100** with allene in the preparation of spiro adduct **103** [46]. (B) The proposed catalytic cycle. (C) Application of phosphine-catalyzed asymmetric [3 + 2] annulation to prepare the chiral adduct **105** with symmetric isoindigo **104** in the formal synthesis of (−)-ditryptophenaline (**12**).

In 2019, Lu and co-workers disclosed a novel chiral-phosphine-catalyzed enantioselective [3 + 2] annulation of allenes and isoindigos to give an enantioenriched annulation adduct bearing vicinal quaternary stereocenters [46] (Scheme 7A). Both symmetric and unsymmetric isoindigos can undergo enantioselective [3 + 2] annulation with an allene and produced a chiral adduct with high yield and high ee value. When unsymmetric isoindigo **100** was used as substrate, enantioselective [3 + 2] annulation with allene **101** in the presence of amino acid-derived bifunctional phosphine **102** produced adduct **103** in 90% yield with 92% ee and 4:1 regioisomeric ratio (rr). The authors suggested that the observed regioselectivity could be rationalized by the proposed catalytic mechanism (Scheme 7B). The phosphine (i.e., PR_3, _**A**) attacks the allene **101** to generate zwitterion intermediate **B**, which is subjected to a less hindered attack by the isoindigo **100**. The oxindole bearing a chlorine atom on isoindigo **100** makes C-3 more electron deficient than C-3’, which results in the regioselective formation of intermediate **C**. Cyclization of intermediate **C** gives **D** and subsequent proton transfer produces isomer **E**. It undergoes elimination to afford the annulation product **103** and the phosphine catalyst **A** is regenerated.

In the same work, Lu and co-workers applied the enantioselective [3 + 2] annulation to complete the formal synthesis of (−)-ditryptophenaline (**12**) [46] (Scheme 7C). The synthesis began with the catalytic asymmetric [3 + 2] annulation of symmetric isoindigo **104** and allene **101** with chiral phosphine catalyst **102** to give spirocyclic adduct **105** in 93% yield with 99% ee. The freshly prepared enantioenriched adduct **105** was subjected to ozonolysis [47] followed by decarboxylation to give bisoxindole **106** in 68% yield over two steps. Conversion of **106** to the corresponding acetal and subsequent allylation afforded **108** in 86% yield over two steps. A two-step synthesis from **108** produced **109**, which was converted to (−)-ditryptophenaline (**12**) by using Overman’s protocol [48].

### Rhodium-catalyzed [3 + 2] cycloaddition

**Scheme 8:**
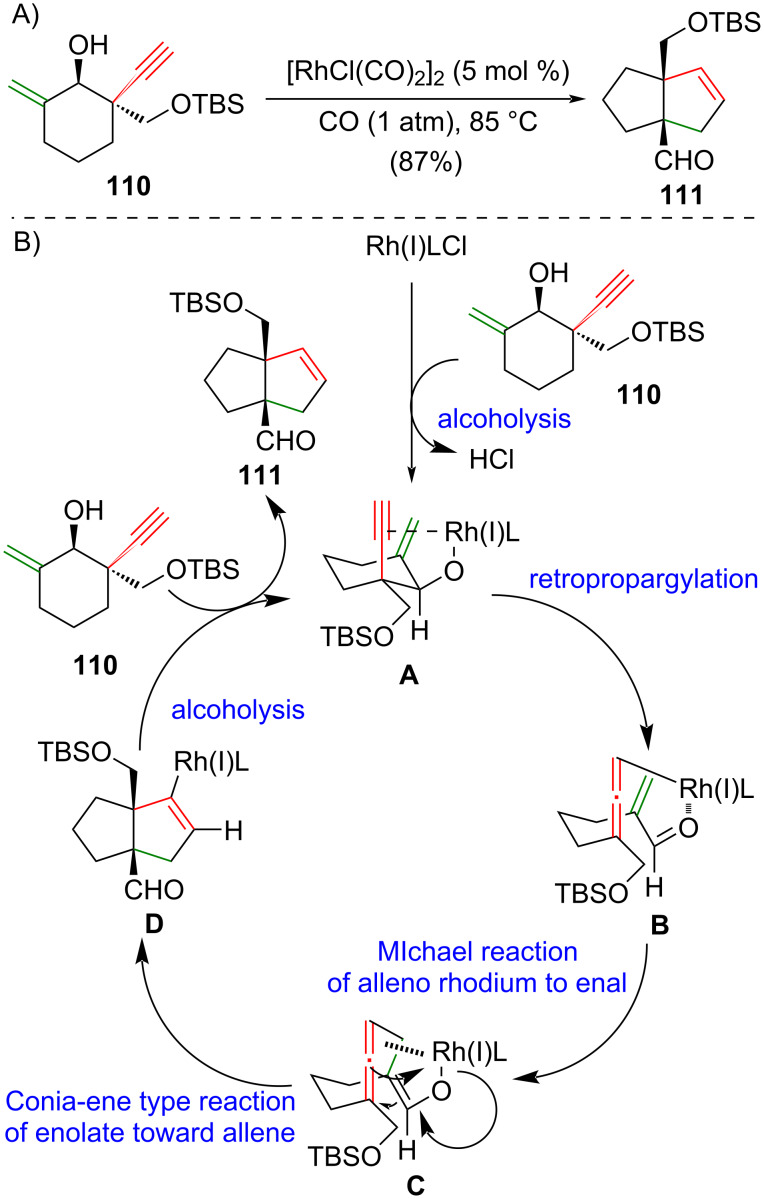
(A) Rhodium-catalyzed intracmolecular [3 + 2] cycloaddition [49]. (B) The proposed catalytic cycle of the reaction.

**Scheme 9:**
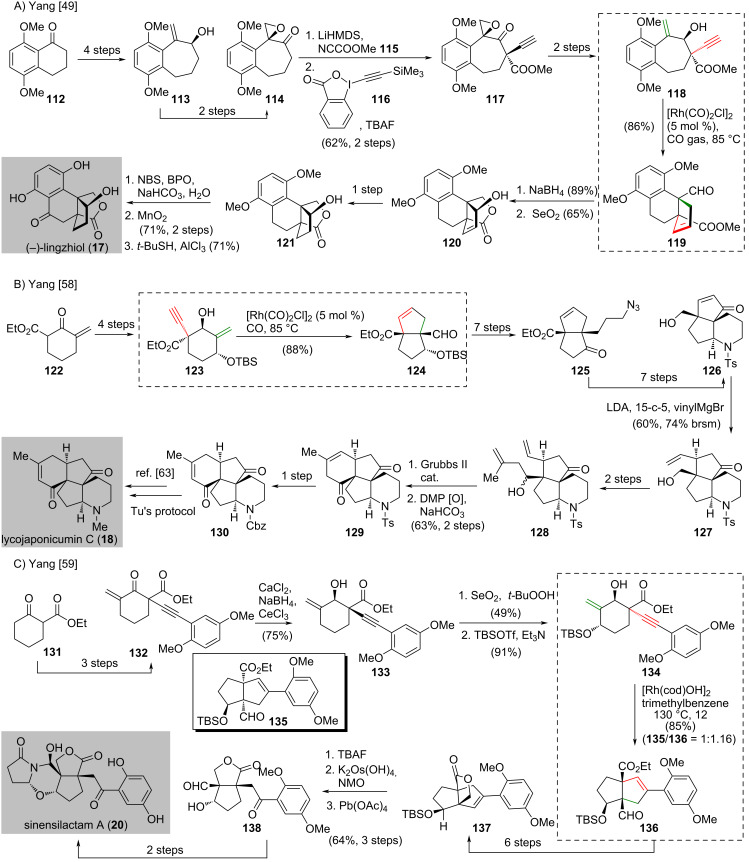
Total synthesis of natural products reported by Yang and co-workers applying rhodium-catalyzed intramolecular [3 + 2] cycloaddition. (A) Synthesis of (−)-lingzhiol (**17**) [49]. (B) Synthesis of lycojaponicumin C (**18**) [58]. (C) Synthesis of sinensilactam A (**20**) [59].

In 2014, Yang and co-workers reported an efﬁcient rhodium-catalyzed intramolecular [3 + 2] cycloaddition of **110** to give [3.3.0] and [3.4.0] bicyclic systems bearing two quaternary atoms at the bridgehead position [49]. For instance, enynol **110** was treated with 5 mol % of [RhCl(CO)_2_]_2_ and carbon monoxide to afford a [3.3.0] bicycle **111** in 87% yield (Scheme 8A). The proposed catalytic cycle of this elegant rhodium-catalyzed intramolecular [3 + 2] cycloaddition begins with the reaction between the rhodium catalyst Rh(I)LCl and alcohol **110** to give complex **A** through alcoholysis [50–51] (Scheme 8B). Rh(I)-mediated retro-propargylation of the homopropargyl alcohol **A** afforded complex **B**. It undergoes an intramolecular Michael addition [52–53] with the allenyl rhodium to the enal and gives the allenyl rhodium species **C**. A Conia-ene-type reaction [54] between the Rhoda-enolate species and the allene of complex **C** produces the desired [3.3.0] bicycle **D**. Protonolysis [55–57] of complex **D** with the alcohol **110** gives bicyclic product **111** and regenerates the rhodium complex **A**. This elegant method has been successfully applied by the same research group in their synthesis of lingzhiol (**17**) [49], lycojaponicumin C (**18**) [58] and sinensilactam A (**20**) [59] (Scheme 9).

The synthesis of (−)-lingzhiol (**17**) was reported by Yang and co-workers in 2014 [49] (Scheme 9A). The synthesis began with the conversion of ketone **112** into alcohol **113** in four steps, which involved a hypervalent iodine-mediated ring expansion [60]. A two-step synthesis from **113** gave epoxide **114**. Epoxide **114** was converted to the corresponding β-ketoester and subsequent treatment with Waser’s reagent **116** [61] afforded alkyne **117** in 62% yield over two steps. Enyne **118**, which was prepared in two steps from **117**, was subjected to rhodium-catalyzed intramolecular [3 + 2] cycloaddition in the presence of carbon monoxide to give tricycle **119** bearing the desired vicinal quaternary carbon stereocenters in 86% yield. Reduction of aldehyde **119** and subsequent transesterification produced a lactone (not shown). It was exposed to SeO_2_ to install the allylic hydroxy group to give **120** in 65% yield. Upon catalytic hydrogenation of **120,** alcohol **121** was formed. This alcohol **120** was subjected to a bromination [62]/oxidation sequence followed by demethylation to produce (−)-lingzhiol (**17**).

After the elegant synthesis of (−)-lingzhiol (**17**) was reported by Yang’s group [49], the same research group disclosed the synthesis of lycojaponicumin C (**18**) [58] and sinensilactam A (**20**) [59] in 2017 and 2018, respectively, featuring the rhodium-catalyzed intramolecular [3 + 2] cycloaddition as the key reaction (Scheme 9B and Scheme 9C). Enyne **123**, which was prepared from enone **122** in four steps, was subjected to the rhodium-catalyzed intramolecular [3 + 2] cycloaddition under carbon monooxide to give the desired bicyclic [3.3.0] aldehyde **124** in 88% yield. A seven-step synthesis from aldehyde **124** gave azide **125**. It was converted to alcohol **126** in seven steps. Alcohol **126** was treated with LDA and vinylMgBr to facilitate a γ-OH directed 1,4-addition [63] to give C-7-vinylated tricycle **127** in 60% yield (74% yield, brsm). A two-step synthesis from **127** produced diene **128**, which was subjected to ring-closing metathesis and subsequent Dess–Martin oxidation to give **129** in 63% yield over two steps. Tetracycle **130**, which was prepared from **129** in one step, was converted to lycojaponicumin C (**18**) via Tu’s protocol [64].

The synthesis of sinensilactam A (**20**) commenced with a three-step synthesis from ketoeseter **131** to give enone **132** [59] (Scheme 9C). Selective reduction of the ketone moiety of **132** was accomplished under Luche’s conditions [65] in the presence of calcium chloride [63] to produce the desired alcohol **133** in 75% yield as a single diastereomer. Allylic oxidation of freshly prepared **133** with SeO_2_ followed by silylation with TBSOTf/Et_3_N afforded enyne **134**. Enyne **134** was subjected to rhodium-catalyzed intramolecular [3 + 2] cycloaddition with a catalytic amount of [Rh(cod)OH]_2_ to produce **135** and **136** in 85% yield in the ratio of 1:1.16. A six-step synthesis from the major product **136** gave lactone **137**. This compond was subjected to successive desilylation, OsO_4_-mediated dihydroxylation and subsequent oxidative cleavage of the C=C double bond with Pb(OAc)_4_ to give ketoaldehyde **138** in 64% yield over three steps. The conversion of **138** to sinensilactam A (**20**) was achieved in two steps.

### Platinum-catalyzed [3 + 2] cycloaddition

**Scheme 10:**
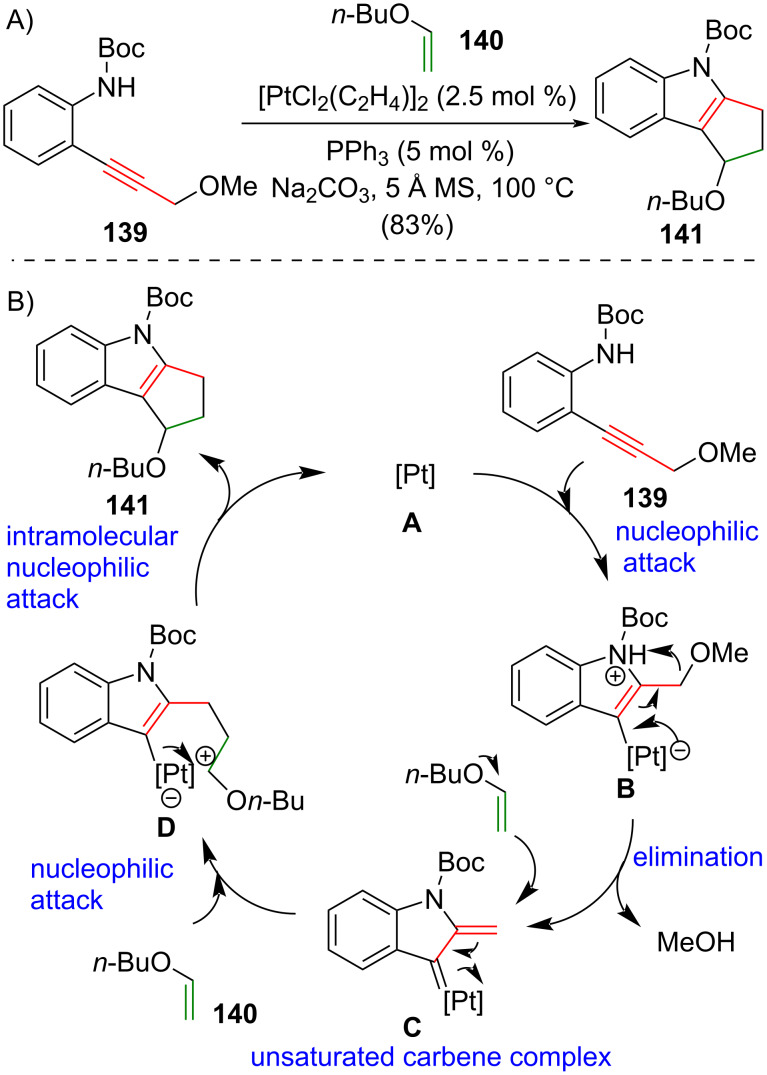
(A) Platinum(II)-catalyzed intermolecular [3 + 2] cycloaddition of propargyl ether **139** and *n*-butyl vinyl ether (**140**) gives tricyclic indole **141** [18,66]. (B) The proposed mechanism.

The platinum-catalyzed intermolecular [3 + 2] cycloaddition of propargyl ether derivatives and vinyl ether producing polycyclic indoles was disclosed by Iwasawa and co-workers in 2011 [18,66] (Scheme 10A). Treatment of Boc-protected aniline **139** and *n*-butyl vinyl ether (**140**) with a platinum(II) catalyst afforded tricyclic indole **141** in 83% yield. The authors suggested that this catalytic [3 + 2] cycloaddition reaction may involve an α,β-unsaturated carbene complex intermediate and a mechanism was proposed (Scheme 10B). An nucleophilic attack of the amine nitrogen onto the alkyne **139** under the effect of activated Pt(II) **A** produces zwitterionic intermediate **B**. Elimination of the methoxy group from zwitterion **B** generates the α,β-unsaturated carbene complex intermediate **C**. **C** is subjected to the nucleophilic attack of *n*-butyl vinyl ether (**140**) and generates alkenyl metallic intermediate **D**. Intramolecular nucleophilic attack onto the oxonium carbon of **D** aﬀords the [3 + 2] cycloaddition product **141** with regeneration of the catalyst **A**.

**Scheme 11:**
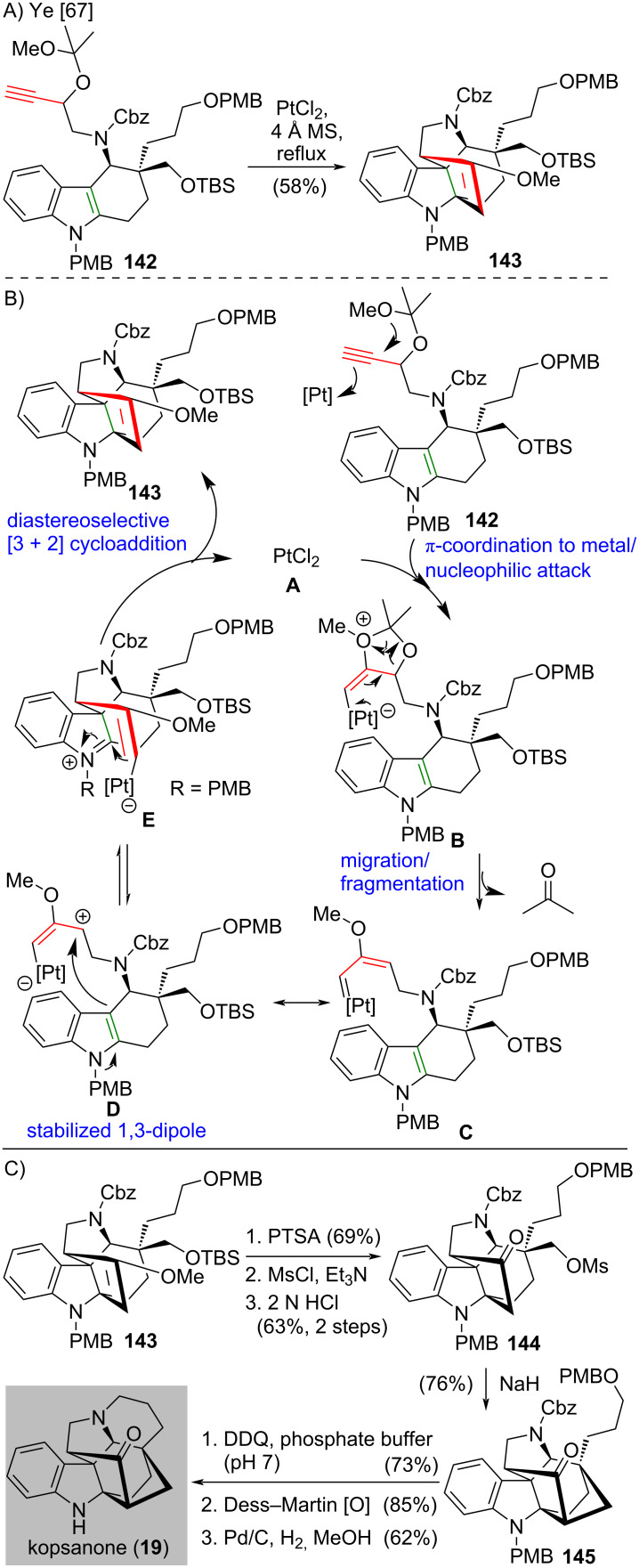
(A) Platinum-catalyzed intramolecular [3 + 2] cycloaddition of propargylic ketal derivative **142** to give indoline **143** [67]. (B) The proposed catalytic mechanism. (C) The completion of total synthesis of kopsanone (**19**).

In 2020, Ye and co-workers used a platinum-catalyzed intramolecular [3 + 2] cycloaddition of a propargylic ketal derivative to complete the total synthesis of Kopsia indole alkaloids [67] (Scheme 11). The platinum-catalyzed intramolecular [3 + 2] cycloaddition of propargylic ketal derivative **142** afforded indoline **143** in 58% yield, which possesses three contiguous stereocenters with vicinal all-carbon quaternary centers. (Scheme 11A). According to the proposed mechanism, coordination of the triple bond of **142** to the electrophilic platinum complex **A** followed by intramolecular nucleophilic attack by the methoxy group gives complex **B** (Scheme 11B). A facile migration–fragmentation process of complex **B** eliminates a ketone through fragmentation and produces metal-carbene intermediate **C**. The freshly prepared metal-carbene **C** is equilibrated to stabilized 1,3-dipole **D**. **D** undergoes a diastereoselective [3 + 2] cycloaddition to give indoline **143** and the active platinum catalyst **A** is regenerated. After the successful preparation of indoline **143**, the synthesis of kopsanone (**19**) is accomplished (Scheme 11C). Indoline **143** was converted to ketone **144** in three steps, which was subjected to a nucleophilic substitution to give the cyclization product **145** in 76% yield. The hexacyclic compound **145** was converted to kopsanone (**19**) in three steps.

### Miscellaneous

**Scheme 12:**
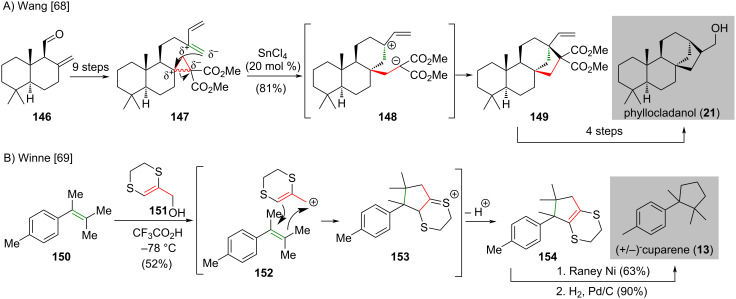
(A) Synthesis of phyllocladanol (**21**) features a Lewis acid-catalyzed formal intramolecular [3 + 2] cross-cycloaddition of cyclopropane 1,1-diesters with alkenes [68]. (B) (5,6-Dihydro-1,4-dithiin-2-yl)methanol **151** used as a versatile allyl-cation equivalent in [3 + 2] cycloaddition in the synthesis of (±)-cuparene (**13**) [69].

In 2012, Wang and co-workers reported a Lewis acid-catalyzed intramolecular [3 + 2] cross-cycloaddition (IMCC) of cyclopropane 1,1-diesters with non-activated alkene to generate bridged [*n*.2.1] carbocyclic skeletons, which is applied to the synthesis of phyllocladanol (**21**) [68] (Scheme 12A). The IMCC precursor **147** was prepared from aldehyde **146** in nine steps. The IMCC precursor **147** underwent an intramolecular cross-cycloaddition catalyzed by tin tetrachloride to give tetracycle **149** in 81% yield. The authors suggested that the intramolecular [3 + 2] cross-cycloaddition of the less-substituted external carbon atom in the C=C double bond results in the formation of the more stable internal carbenium (i.e., **148**) and promotes IMCC to give the bridged [3.2.1] octane **149**. The transformation of **149** to phyllocladanol (**21**) was accomplished in four steps.

In 2016, Winne and co-workers reported that (5,6-dihydro-1,4-dithiin-2-yl)methanol (**151**) can be served as a allyl-cation equivalent for the [3 + 2] cycloaddition and was applied in the synthesis of (±)-cuparene (**13**) [69] (Scheme 12B). An intermolecular [3 + 2] cycloaddition of tetrasubstituted alkene **150** and the dhdt-2-methanol reagent **151** under the effect of trifluoroacetic acid produced adduct **154** in 52% yield. The authors identified that the cyclic nature of the dhdt-2-methanol reagent **151** is essential for the cycloaddition to take place. The use of noncyclic analogues did not give the cycloaddition product. It is suggested that the restricted rotational freedom of **151** and the related enforced conjugation of the sulfur lone pair may block certain undesired cation reactions. Cycloaddition product **154** was subjected to the hydrodesulfurization with Raney nickel as catalyst and subsequent catalytic hydrogenation produced (±)-cuparene (**13**) in 90% yield.

### All-carbon [3 + 2] annulation in natural product synthesis

**Scheme 13:**
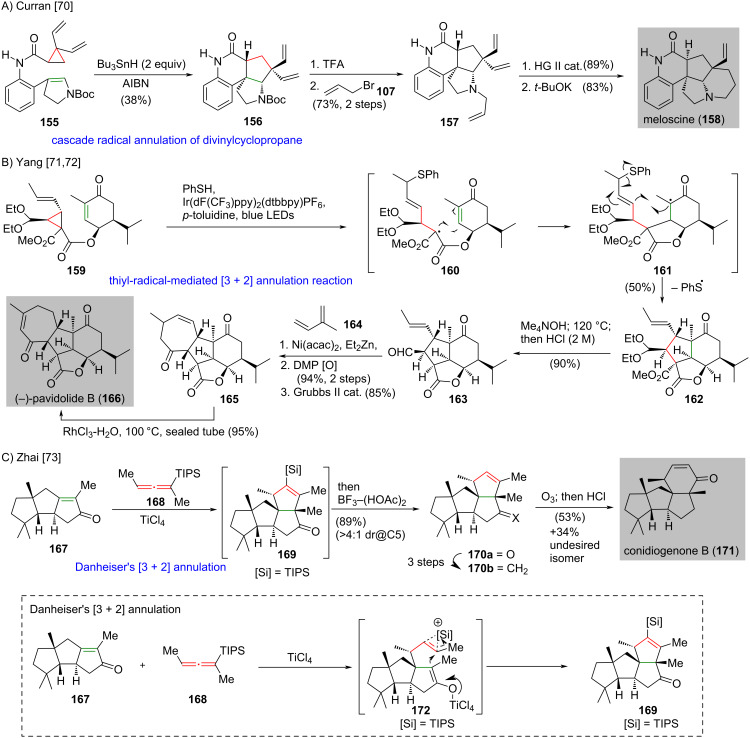
The recent advances of [3 + 2] annulation in natural product synthesis. (A) The preparation of meloscine (**158**) features a cascade radical annulation of divinylcyclopropane [70]. (B) Thiyl-radical-mediated [3 + 2] annulation reaction realizes the synthesis of (−)-pavidolide B (**166**) [71–72]. (C) A Danheiser’s [3 + 2] annulation en route to conidiogenone B (**171**) [73] (inset, the suggested mechanism based on Danheiser’s proposal disclosed in 1981 [74].)

The all-carbon [3 + 2] cycloaddition demonstrated the ability to assemble intricate polycyclic structures in the synthesis of complex natural products. Besides the all-carbon [3 + 2] cycloaddition reactions and the corresponding applications described above, the all-carbon [3 + 2] annulation, which undergoes other possible mechanistic pathways other than cycloaddition, proved its usefulness in forging highly-substituted five-membered carbocycles. These reactions have been applied successfully in the synthesis of complex natural products. In 2011, Curran and co-workers reported the synthesis of meloscine (**158**) featuring a tandem radical cyclization of a divinylcyclopropane [70] (Scheme 13A). Slow addition of tributylstannane and AIBN to a refluxing solution of cyclopropane **155** afforded **156** in 38% yield. It was subjected to cleavage of the Boc group followed by *N*-allylation to give **157** in 73% yield over two steps. A ring-closing metathesis of freshly prepared **157** was effected by the second generation Hoveyda–Grubbs (HG II) catalyst and subsequent base-promoted epimerization produced meloscine (**158**) in 83% yield.

In 2017, Yang and co-workers disclosed the synthesis of (−)-pavidolide B (**166**) by using a thiyl-radical-mediated [3 + 2] annulation reaction to create four contiguous stereocenters on tricycle **162** in one step [71–72] (Scheme 13B). Exposure of ester **159** to PhSH [75], *p*-toluidine and a catalytic amount of Ir(dF(CF_3_)ppy)_2_(dtbbpy)PF_6_ under the irradiation of blue LED light [76–77] afforded tricycle **162** in 50% yield. The authors suggested that this process involves an intramolecular 5-*exo-*conjugated addition of a radical on **160** to the enone and produces **161**. The newly formed **161** was subjected to 5-*exo* radical addition to the allyl sulfane and subsequent loss of a thiyl radical produces **162**. A successive hydrolysis/decarboxylation upon heating and cleavage of acetal on **162** afforded aldehyde **163** in 90% yield. Coupling of aldehyde **163** and isoprene (**164**) with Ni(acac)_2_ and diethylzinc [78] and then Dess–Martin oxidation gave a diene (not shown, 94% yield over two steps), which was subjected to ring-closing metathesis to give enone **165** in 85% yield. Isomerization of the freshly prepared **165** to more stable α,β-unsaturated enone with RhCl_3_ [79] afforded pavidolide B (**166**) in 95% yield.

The synthesis of (−)-conidiogenone B (**171**) featured a Danheiser’s [3 + 2] annulation [74,80] and was reported by Zhai and co-workers in 2020 [73] (Scheme 13C). Treatment of tricycle **167** with allene **168** in the presence of TiCl_4_ gave the desired **169** carrying two vicinal quaternary carbons. A one-pot desilylation of the newly formed **169** with a trifluoride–acetic acid complex produced the tetraquinane **170a** in 89% yield with a 4:1 dr. The conversion of the freshly prepared ketone **170a** to **170b** was achieved in three steps. Ozonolysis of the C=C double bond of **170b** gave a keto aldehyde (not shown), which was subjected to an acid-mediated aldol reaction to give conidiogenone B (**171**) in 53% yield. The undesired isomer with β,γ-C=C double bond (not shown) was formed in 34% yield and can be isomerized to the more stable α,β-unsaturated enone to afford conidiogenone B (**171**) in 32% yield upon treatment with RhCl_3_ in microwave.

The reaction mechanism of Danheiser’s [3 + 2] annulation is shown according to the Danheiser’s proposal [74] (Scheme 13C, inset). Initial complexation of the α,β-unsaturated ketone **167** and titanium tetrachloride produces an alkyoxy allylic carbocation (not shown). This carbocation is subjected to a regiospecific electrophilic substitution of allene **168** to generate a vinyl cation **172**, which is stabilized by an adjacent carbon–silicon bond. The 1,2-shift of the silyl group in **172** produces an isomeric vinyl cation, which is intercepted by the titanium enolate and results in the new C–C bond formation to give the five-membered carbocycle **169**.

## Conclusion

The all-carbon [3 + 2] cycloaddition, together with the [3 + 2] annulation, continue to be an attractive class of reactions for the synthesis of highly-substituted and stereo-congested five-membered carbocycles. Also, one or more quaternary carbons can be created in a single reaction making this class of reactions appealing to complex natural product syntheses. This review outlines the development of the all-carbon [3 + 2] cycloaddition and its application in natural product synthesis reported from 2011–2020 (inclusive). The intermolecular all-carbon [3 + 2] cycloaddition offers a facile approach to install functionalized five-carbon carbocycles, including fused-rings (e.g., longeracinphyllin A (**10**)) and/or spiro-ring (e.g., marcfortine B (**8**)), at later stage of the synthesis without the need of pre-installation of necessary functional groups as a reaction precursor, for instance, ring-closing metathesis, intramolecular aldol condensation, and others.

One major issue that still needs to be addressed is the selectivity of the all carbon [3 + 2] cycloadditions, which are usually under substrate-control. Remarkable innovation of the stereoselective palladium-catalyzed trimethylenemethane cycloaddition reported by Trost’s group, which makes use of catalytic amounts of palladium and chiral phosphine ligand **74**, was applied successfully in the enantioselective synthesis of marcfortine C (**9**, Scheme 4B). Another brilliant example is the development of a chiral-phosphine-catalyzed [3 + 2] annulation reported by Lu in 2019, in which the chiral phosphine catalyst confers high stereocontrol on the formation of a spiro adduct bearing two vicinal all-carbon quaternary stereocenters (Scheme 7). We believe that the enantioselective all-carbon [3 + 2] cycloaddition provides a new strategy for the preparation of sp^3^-carbon-enriched complex scaffolds [81–82] for biological studies and potential new drug development.

The all-carbon [3 + 2] cycloaddition is undoubtedly an efficient synthetic transformation that creates two C–C bonds in a single reaction. However, the prior protection of the reactive functional groups, such as the hydroxy and amino groups, are still necessary for most of the all-carbon [3 + 2] cycloaddition reactions. We predict that further development of the all-carbon [3 + 2] cyclization with the reactive functional groups’ compatibilities and/or without the use of protecting groups [83–84] can improve the synthetic efficiency and make this class of reactions more attractive to the synthetic scientist for applications. Lastly, we anticipate that the all-carbon [3 + 2] cycloaddition will gain further attention from the synthetic community, including scientists from academia and pharmaceutical industry, for methodic innovation and the efficient synthesis of biologically important natural products.
